# The Anchored Testimony Calendar: A Black Feminist Method for Retrospective Behavioral Health Inquiry with Justice-Impacted Young Adults and Caregivers

**DOI:** 10.1080/08964289.2026.2683391

**Published:** 2026-10-21

**Authors:** Erin Michelle Turner Kerrison, Megan S. Irgens, Cynthia Valencia-Ayala, Johanna B. Folk, Salam Ayana, Akilah Dulin, Sophia Garcia-Meza, Brandon David Lewis Marshall, Elizabeth McBride, Jeanne McPhee, Larry Brown, Marina Tolou-Shams

**Affiliations:** a University of California, Berkeley; b University of California, San Francisco; c Tulane University; d Brown University; e Rhode Island Hospital and Brown University

**Keywords:** Anchored Testimony Calendar, Black feminist epistemology, equity-oriented research, justice-impacted young adults (juvenile justice), life course methodology, qualitative behavioral health research, retrospective interview, trauma- and violence-informed care (TVIC)

## Abstract

Retrospective behavioral health instruments calibrated for chronological precision and discrete-symptom reconstruction can fail people whose lives have been organized by structural racism, sexism, and economic inequality. We describe the Anchored Testimony Calendar (ATC), a Black feminist adaptation of the event history calendar developed within the Epidemiological Project Involving Children in the Court (EPICC), a longitudinal study of justice-impacted young adults and caregivers launched in 2014. ATC pre-populates an interviewer-facing four-domain grid—behavioral and psychiatric health, education, family context, and juvenile legal system involvement—with the participants’ own survey responses from seven adolescent assessment waves, anchoring recall to participant-identified transitions rather than calendar dates. Grounded in Black feminist epistemological commitments—lived experience, dialogue, and an ethic of care that refuses extraction—ATC privileges contextual fidelity and cross-domain coherence over chronological precision. From a target of 50 dyads at the young adult follow-up, 38 young adults and 19 caregivers completed an ATC-guided interview before federal funding was terminated. Four vignettes (two caregiver, two young adult) illustrate the instrument at work, and surface structural conditions as participants name them. This paper documents science that federal funding cuts interrupted. ATC is offered as a usable tool, an open methodology, and a record of what behavioral medicine stands to lose.

## Introduction

Adolescent behavioral health does not unfold in isolation, and it does not unfold on a clean timeline. For young adults and their caregivers who are in contact with the legal system (herein referred to as “justice-impacted”), substance use, psychiatric symptoms, school disengagement, and family conflict are forged inside unequal environments shaped by housing instability, public divestment, and chronic institutional surveillance. Most retrospective survey instruments seek to isolate individual symptoms and demand precise dates, treating memory as a tidy archive of discrete behaviors. For people whose lives have been saturated by the criminal legal system, instruments built on those assumptions can mistake compliance for accuracy and silence for absence—not because they are wrong about what they measure, but because they were calibrated for different epistemic purposes than the present study requires.

Structural racism, sexism, and economic inequality are not backdrops to behavioral health development; they are the medium through which risk and protection accumulate across the life course. Ecodevelopmental Theory provides a multilevel framework for organizing micro-, meso-, exo-, and macro-system influences on adolescent risk behavior,^1,2^ and Krieger’s Ecosocial Theory extends that lens by naming embodiment—the biological and social process through which structural discrimination becomes inscribed in bodies and trajectories—as the core object of study.^3^ Intersectionality refuses the additive logic that would treat race, gender, and class as parallel and independent domains, insisting they are co-constitutive at every level of analysis.^4,5^ Trauma- and violence-informed care offers part of the methodological answer by centering an individual’s agency, emotional safety, and contextualized meaning-making.^6,7^ But trauma-informed framing without trauma-informed instrumentation leaves the field with well-intentioned interviewers using tools designed for different epistemic purposes than the lives in front of them demand.

The Anchored Testimony Calendar (ATC) is a Black feminist health-and-justice research instrument for retrospective life-course inquiry with people whose memories were formed under conditions of structural inequity. ATC operationalizes these commitments as method, translating the Ecodevelopmental–Ecosocial–intersectional frame from theoretical stance into interview practice. Throughout this paper, “ATC” refers to the interviewer-facing calendar instrument itself—the pre-populated, four-domain grid that scaffolds an interviewer’s questions across the life course—and the semi-structured life course interview during which it is used is described as the ATC-guided interview. ATC is grounded in three commitments from Black feminist epistemology: lived experience as a criterion of meaning, dialogue as the mechanism through which knowledge claims are assessed, and an ethic of care that refuses extraction.^8^ ATC is grounded equally in intersectional public health scholarship that holds microlevel experience and macrolevel inequality in view simultaneously and treats historically marginalized populations as the proper starting point of inquiry.^4,9^

A note on “testimony” is warranted, because the word does different work in different traditions. In legal contexts, testimony is most often compelled—subpoenaed, cross-examined, and shaped by institutions that have rarely served the people made to speak in them. In the Black feminist tradition, testimony names the practice of speaking lived experience as a criterion of meaning, an epistemic act that counters erasure and centers the knowledge claims of people whose accounts have been systematically disbelieved or extracted without care.^8,10^ We use the term in that Black feminist sense throughout, and we name the instrument the Anchored Testimony Calendar precisely because the people at the center of this study have been subjected, repeatedly and across institutions, to the compelled-testimony version. ATC is built to break with that extractive logic.

We developed ATC within the Epidemiological Project Involving Children in the Court (EPICC), a longitudinal study of justice-impacted youth and caregivers launched in 2014.^11^ As the study moved from adolescent assessment into a young adult re-engagement window roughly a decade later, a qualitative component was added to provide contextual, sequenced, multi-domain accounts of how structural forces are experienced and made meaningful by the people moving through them. This article describes ATC’s methodological foundation and illustrates the approach through four vignettes (two caregivers, two young adults) drawn from the qualitative re-engagement wave.

## Methods

### Study design and theoretical framework

EPICC began in 2014, when participating youth were on average 14.5 years old, with a young adult re-engagement window roughly a decade after first juvenile court contact.^11^ EPICC integrates four data sources—survey assessment, the ATC-guided narrative interview, statewide behavioral health services utilization records, and population-level indicators of structural racism, sexism, and economic inequality. Ecodevelopmental Theory and Krieger’s Ecosocial Theory ground the work, and an intersectional analytic lens drawn from Black feminist thought shapes how those frameworks are interpreted and applied. The qualitative arm was designed to enact that commitment, and the ATC is the methodological instrument built to do so. All procedures were approved by the University of California, San Francisco Institutional Review Board (Protocol #13–11738).

### Sample and recruitment

The parent study originally enrolled 401 youth ages 12–18 and an involved caregiver between 2014 and 2016, with adolescent and caregiver survey waves completed every four months across seven timepoints (T1 through T7) over two years. This article reports on the eighth wave (T8), which began in 2022 as part of a follow-up into young adulthood and was the wave at which participants completed a re-enrollment survey assessment and were invited to complete their first ATC-guided interview. Subsequent annual timepoints (T9 through T11) extend the qualitative re-engagement and continue paired survey assessment.

Each T8 ATC was pre-populated with the adolescent survey or caregiver assessment data recorded during the T1 through T8 data collection points. To avoid producing a sample limited to the most stable or easily located families, the team attempted to contact all 397 individuals who had previously consented to follow-up. Every eligible young adult and caregiver was invited to participate, regardless of changes in residence, family structure, or system involvement, and participants could enroll independently when their caregiver could not be reached. That recruitment decision is itself a Black feminist methodological commitment, a deliberate departure from producing a sample weighted toward families with fewer system entanglements, since those are the families whose accounts have most often been cut from the behavioral health record. From a target of 50 dyads at T8, 38 young adults and 19 caregivers completed an ATC-guided interview, including 15 young adult and caregiver dyads.

### Rationale for the Anchored Testimony Calendar

ATC’s contribution to the field is the design of the instrument, not the invention of a new interview protocol. The interview guide remains a life course interview; the ATC is what allows that interview to operate cross-domain and anchored to participant-identified transitions without requiring chronological precision from the people speaking.

The ATC was designed for recall across long, nonlinear developmental periods. Young adults and caregivers who reengaged nearly a decade after their first study assessment were asked to reflect on years marked by residential instability, shifting school placements, family disruption, health crises, and variable institutional visibility—conditions under which recall centers on transitions and contextual shifts rather than sequential enumeration. That insight is not new to the calendar method,^12,13^ and the first author has previously used calendar-anchored retrospective interviewing with a longitudinally reengaged sample of justice-impacted women, where participant-anchored life events functioned more reliably than chronological prompting for memory organized around institutional contact.^14^ ATC builds on that empirical foundation by operationalizing those practices inside an explicit Black feminist epistemological frame, where lived experience is itself a criterion of meaning and a participant’s organization of memory deserves to be the unit of analysis rather than an obstacle to a researcher’s preferred timeline.^8^ Anchoring recall to participant-identified transitions—school changes, household composition shifts, changes in supervision—lets interviewers support temporal orientation while preserving narrative flow.

The calendar functions as a flexible scaffold that holds multiple life contexts in view at once. Its rows represent four ecological domains—behavioral and psychiatric health, education, family context, and juvenile legal system involvement—selected not to mirror primary outcomes but to model the contexts in which those outcomes emerge. Its columns map to the seven adolescent timepoints (T1 through T7), and each cell is pre-populated with the contemporaneous survey responses offered by the young adult and caregiver during those adolescent years. See Appendix A for the full measure list and Figure 1 for the conceptual structure.

### Interviewer training and fidelity

ATC interviews require interviewers who can hold both structure and relational attunement simultaneously. Interviews were conducted by two doctoral-level researchers, the first and third authors, both with prior qualitative interviewing experience and shared commitments to trauma-informed, violence-informed, justice-attentive research. This includes a Black feminist unwillingness to treat a participant’s testimony as data to be mined.

ATC interviewer capacity developed iteratively across the study team through a combination of shadowing, strucutred review, and ongoing case discussion. The data-entry protocol was developed and led by the second author. The third author was onboarded into ATC interviewing through shadowing of live interviews led by the first author, structured review of ten prior interviews, and ongoing case discussion between the two interviewers about pacing, prompt selection, and interpretive choices. When scheduling required handoffs between longitudinal waves, preparation included structured review of the other interviewer’s audio and transcripts, a cross-review that served both as preparation and as a between-interview fidelity check. A structured training and fidelity protocol is in development and will be reported separately.

### ATC-guided interview procedures

Each young adult and caregiver completed an approximately 90- to 120-minute, semi-structured ATC-guided life course interview. Interviews opened with grounding prompts that located them in a developmental period rather than on a calendar date—for example, “Tell me what life felt like around the time you first joined the study”—easing them into narrative reflection before the interviewer guided them across the calendar’s domains. The guide ensured structured coverage of behavioral and psychiatric health, education, family context, juvenile legal system involvement, substance use, relationships, and access to supports. Questions about institutional racism, gender and sexual marginalization, and economic inequality were embedded within their storytelling rather than asked as a discrete module, a Black feminist methodological choice that rejects additive logic and lets context-level identification emerge on the participant’s own terms.^4^

Trauma surfaced naturally in many interviews, but trauma narration as practiced in clinical protocols such as Narrative Exposure Therapy or Trauma-Focused Cognitive Behavioral Therapy is not part of ATC.^15,16^ Interviewers avoided soliciting sensory detail or exhaustive recounting of traumatic events and encouraged participants instead to describe meaning, context, and consequences. Interviewers redirected when participants began describing acute trauma in sensory or sequential detail beyond what would serve their comfort or the methodological purpose of the interview. Interviewers normalized stepping back, offered pauses, and made clear that they were free to skip a domain or return to it later. Mandated-reporting disclosures were handled using transparent, person-centered protocols established at consent.

### Use of the ATC during interviews

The ATC was interviewer-managed and never shown to the participant. Showing the grid risks shifting the participant into task completion and reproducing the dynamic that justice-impacted populations have already encountered across institutions, in which their own data are handed back as instruments of accountability rather than as material for their own meaning-making. The grid shapes what is asked, but it never appears in the room.

The grid was pre-populated with the participant’s own T1 through T7 responses, including items from the DSM-5 Cross-Cutting Symptom Inventory, the Behavior Assessment System for Children (BASC), the Adolescent Risk Behavior Assessment (ARBA), juvenile legal system involvement items, and education and housing modules.^17–19^ Pre-populating with the young adult’s or caregiver’s own prior responses, rather than with researcher-imposed categories, returns the individual’s account to them rather than asking her to confirm a frame she did not author. Interviewers used those entries as gentle prompts—for example, “Was that around the time of the move?”—to help them locate memories within familiar developmental periods. Table 1 illustrates the prompt families and their narrative functions; each vignette below is keyed to one or more prompt families in Table 1.

### Testimony in practice

What follows is not a findings section. Each participant decided what mattered, what to name, and what to leave alone. The four accounts below are drawn from T8 ATC-guided interviews, two with caregivers and two with young adults, and they show how the ATC operates in the room across different narrative positions rather than compare cases or build claims about substance use, school discipline, or legal contact. All names are pseudonyms.

### Elena (caregiver)

Elena set the terms of her interview in the first minute. “These are wounds, not scars… you’re asking this woman to go back to a place I don’t think you’ll ever leave.” What she was describing was not a closed chapter but an open one she had been living inside for years.

What she remembered first was not a sequence of events but a feeling—family deaths, a flat affect she could not lift, parenting moments she would replay later and not recognize herself in. The episodes did not arrive in order. They arrived together, fused.

Elena’s ATC, held by the interviewer, carried entries Elena herself had reported spanning the seven adolescent waves—moves, household shifts, school transitions, behavioral concerns the schools had flagged. The interview opened with the *Opening / Grounding* prompt in Table 1—“… can you tell me what life felt like around this time?”—and that was the question that gave Elena room to choose what came first. From there, the interviewer leaned on *the Participant Control Check* and *Distress Monitoring* prompts in Table 1 to keep the pacing in Elena’s hands. “They see you as troubled before they see you as hurting,” she said, naming the racialized and gendered misread she had lived under as a Black mother trying to advocate for a child the schools had already labeled. That sentence was not pulled out of her; it surfaced because the question that came before it gave her room to say it.

By the time she paused, Elena had separated grief from parenting strain from institutional misinterpretation, three threads she had long carried as one. She did not need the calendar to do that work for her. She needed the conversation to be patient enough for her to do it herself.

### Darlene (caregiver)

Darlene came to the interview wanting to talk about her daughter. Her daughter’s disappearance at age fourteen had been the worst stretch of Darlene’s life, and in her telling the months came out the way they had lived in her—“fear, long nights searching, unanswered calls to police”—without seams.

The interviewer did not ask Darlene to date anything. The grid carried Darlene’s entries from across the adolescent waves—her daughter’s school attendance, who was in the household and when, juvenile court contacts logged and not. When Darlene started describing her daughter slipping out at night, the interviewer used the *Temporal Orientation (Non-Date-Based)* prompt in Table 1—“Was that before or after the move with your grandparents?”—and the *Clarifying Collapsed Memorie*s prompt in Table 1—“… can we slow that down and look at one piece at a time?”

The seams started to show. There had been a stretch when her daughter was newer to a school and falling away from it, a different stretch when an older girl Darlene had warned her daughter about kept reappearing, and a stretch after that when Darlene was calling police and being told she was calling too much. “They told me I was calling too much,” she said. “Maybe she didn’t run away.” Said in real time, the line was an indictment. Said in the calendar’s slower light, it became a pattern—every dismissal had landed on a week when something else had already been falling apart.

What the ATC did for Darlene was the labor of letting an undifferentiated emergency become a sequence of separate moments she could see clearly enough to be angry about properly.

### Jordan (young adult)

Jordan started with a sentence that did most of the work for him. “They decided who I was before I even walked into the room.” The rest of the interview filled in what they had decided and what it had cost.

His memories of school did not come in order, and he was clear that they had never been in order. Being moved into a different classroom without explanation, getting written up while another kid didn’t, a counselor who looked past him to whoever was sitting next to him—these were not events for Jordan. They were a temperature. Working from Jordan’s adolescent-wave entries—placements, disciplinary contacts, the year he was switched into special education—the interviewer drew on the *Cross-Domain Linking* prompt in Table 1—“What else was going on then— at school, at home, or with the court?”—and closed with the *Closing / Integration* prompt in Table 1—“Looking back, how do you see these experiences fitting together now?”

The questions moved across what Jordan had reported years earlier, and his answers started to move too. He noticed, mid-sentence, that the year he was reclassified academically was the year he stopped raising his hand, and the year he stopped raising his hand was the first year he got into trouble outside of school. He had not connected those before—not because the connection was hidden, but because no one had asked him in a way that let all three be true at once. The interviewer’s questions could move across rows of his life because the ATC in front of her did.

### Khalil (young adult)

Khalil told the story of the night the way they had told it to themselves for years—it had changed everything. “Everything became a violation,” they said. After the incident there was probation, lost shifts, an anxiety that wouldn’t let up.

The interviewer opened with the *Eliciting Anchoring Events* prompt in Table 1—“When you think back over those years, what stands out as a turning point or a period that really shaped things for you?”—which let Khalil name the night themself. She then used the *Substance-Use Contextualization* prompt in Table 1—“How did alcohol or other substances fit into your life during that period?”—to set the night against what else had been moving in their life. Khalil’s adolescent-wave entries showed an eviction notice the same year and an elevated depressive-symptom flag in that stretch, so the interviewer asked about work that summer, about whether they were still at their mother’s. None of those questions were about the night itself.

What came back, slowly, was that the night had not started anything. It had landed inside of a longer crisis that had no start date. Khalil’s mother had been working a shift that ran late. The apartment had been one notice away from being lost since the spring. The depression Khalil had been carrying since middle school was at one of its worse stretches. “They were already watching us,” Khalil said, and meant the apartment, and meant the year, and meant the surveillance that had organized both.

### Thematic synthesis

Throughout the four encounters, the ATC did the same kind of work at four different paces. Memory that had arrived fused became narratable and patterned rather than singular. The pattern emerged not because the interviewer told young adults or caregivers what was connected, but because the calendar’s pre-populated rows gave the interviewer the scope to move between domains as their own narratives moved. The structural framings—“they see you as troubled before they see you as hurting,” “they were already watching us”—were named by these experts, not inferred by the team.^10,20^ What the four vignettes share is a demonstration of how a Black feminist methodological commitment, in which testimony is the focal starting point of inquiry, can be built into instrument design rather than declared as analytic posture.^20,21^

## Discussion

ATC was developed to take the cross-domain, structurally shaped, non-linear character of justice-impacted young adults’ and caregivers’ lives seriously at the level of the instrument itself. Elena, Darlene, Jordan, and Khalil arrived with memories that were fused, blurred, or pre-organized around an institutional misread, and the calendar gave the interviewer enough cross-domain scope and enough pacing control to let those memories become legible on the participant’s own terms. ATC did not extract sequence from chaos. It opened space for them to lay down their own sequence, as the experts on their own lives.

ATC’s signature contribution is a calendar instrument designed inside a Black feminist epistemological commitment rather than fit to one after the fact. The Life History Calendar, the Timeline Followback, and the Event History Calendar all share a recognition that life events anchor recall better than dates do,^12,13^ but each was built for chronological precision and discrete-behavior reconstruction. ATC privileges contextual fidelity and cross-domain coherence instead, and accepts that for people whose lives have been shaped by institutional surveillance, those goals frequently conflict. The method is not a low-burden alternative. The burden it does carry—interviewer skill, longitudinal preparation, and epistemological accountability to the populations it engages—is the burden the method demands.

A second contribution concerns how Black feminist epistemology operates inside the instrument rather than around it. Public health methodological writing has often drawn on Black feminist and intersectional scholarship at the level of analytic framing, while leaving the instruments of data collection untouched.^4,9,22,23^ ATC was designed to dissolve that split. Lived experience as a criterion of meaning shapes the anchor structure. Dialogue as the assessment mechanism shapes the prompt families and the pacing checks. The ethic of care that refuses extraction shapes the interviewer-held grid, the redirection of acute trauma narration, and the recruitment commitment to families with the most system entanglement rather than the least.

That commitment is embedded in a research program the federal government tried to end. The grant supporting EPICC’s qualitative re-engagement was terminated in February 2025 on the agency’s stated grounds that research framed around “amorphous equity objectives” no longer effectuates priorities, and reinstated later that year after administrative challenge.^24^ The T8 interviews reported here had been completed three years earlier. What the cohort absorbed was the closeout-and-restart—the abrupt termination and eventual reinstatement—of a study built around its longitudinal continuity. We name that here not as a procedural footnote but because the methodological commitments this paper has described are the ones the agency-priorities clause was wielded against. The instrument the field most needs is the one most legible to administrative power as expendable.

ATC is offered as a usable tool, not as a closed methodology. Interviewer training and fidelity remain provisional, and the structured curriculum is in development. Interviewer continuity within longitudinal dyads is preferred but not always operationally possible, and the cross-review fidelity practice requires more rigorous evaluation. The vignettes here come from a single longitudinal study and a single qualitative wave; ATC’s portability into other populations and other behavioral health questions remains an empirical question. Future work will examine how ATC narrative data integrate with the EPICC’s full mixed-methods architecture: longitudinal quantitative survey spanning T1 through T11, statewide behavioral health utilization records now capturing participants as young adults, and population-level structural indicators.

## Conclusions

ATC was built around a recognition the team carries from the parent study. The young adults and caregivers who entrust their accounts to this work are extraordinary readers of the institutions they have moved through—analysts and theorists of those institutions in the fullest sense, in the tradition Audre Lorde named when she insisted that the people most marked by structural violence are the ones whose theorizing the field most needs and most often refuses.^25^ Their insight into how systems actually work is the kind of knowledge a calendar has to be designed to receive rather than to manage. ATC is one attempt to build an instrument adequate to that work.

The four vignettes—Elena, Darlene, Jordan, and Khalil—show the calendar at work in four different rhythms. What ATC contributed was not analysis but room. Room for a mother to separate grief from parenting strain from the racialized misread that had collapsed them into one. Room for a mother to move through a crisis account slowly enough that institutional disbelief became visible. Room for a young man to notice that the year he was reclassified academically was the year he stopped raising his hand. Room for a young adult to locate the night that “changed everything” inside a longer landscape of pressure that had already been there.

ATC is offered to research and clinical communities as a usable tool and as an open methodology. It is portable beyond the parent study and beyond the populations it was first built for—to reentry research, perinatal and maternal health, child welfare, opioid epidemiology, HIV care continuum work, and trauma recovery research that requires sequence and meaning without re-traumatization. The method is teachable, and ATC’s training and fidelity protocol is being formalized so that interviewers across career stages can adopt it inside trauma- and violence-informed research practice. We expect ATC to be refined by the research communities who take it up and, most importantly, by the young adult and caregiver communities who continue to teach the field what their accounts require. The instrument’s worth will be measured by whether it continues to make room—for testimony in the Black feminist sense, and for the people whose theorizing the field has too often mined and too rarely honored.

## Supplementary Material

Supp 1

Supplemental data for this article can be accessed online at https://doi.org/10.1080/08964289.2026.2683391.

## Figures and Tables

**Figure 1.**
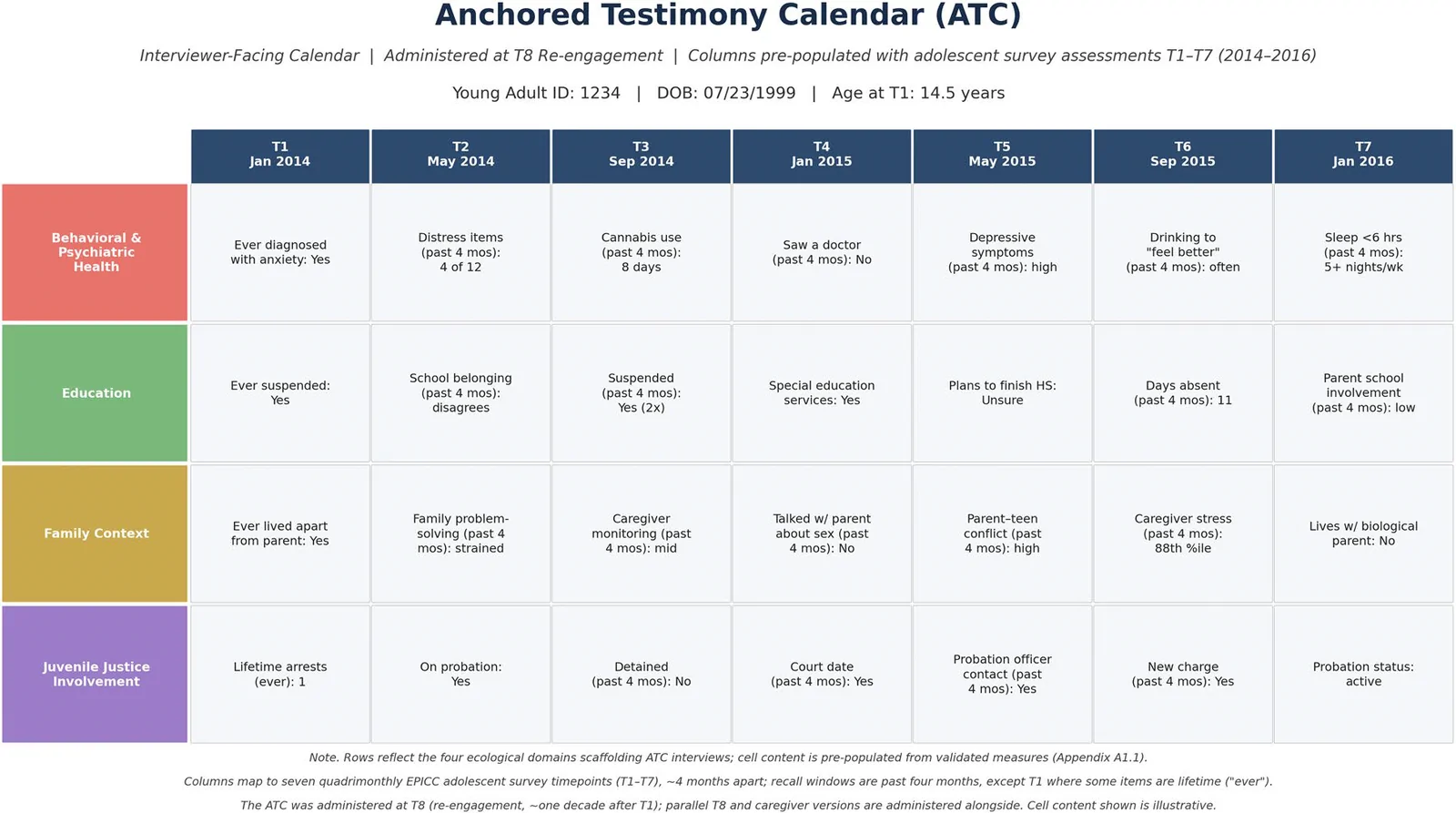
Conceptual structure of the Anchored Testimony Calendar (ATC).

**Table 1.** Interviewer prompts and functions in ATC-guided life course interviews.

	*Anchored Testimony Calendar (ATC) \ EPICC T8 (Interview Wave 1)*		
	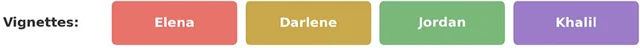		
Interview Phase	Interviewer Prompt (Examples)	Function in ATC Interview	Vignette
Opening / Grounding	*“Before we get into details, can you tell me what life felt like around this time?”*	Eases the participant into narrative reflection; supports emotional regulation and participant control before domain-specific recall begins.	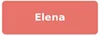
Eliciting Anchoring Events	*“When you think back over those years, what stands out as a turning point or a period that really shaped things for you?”*	Invites participant-defined anchoring events without imposing milestones or chronology.	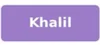
Temporal Orientation (Non-Date-Based)	*“Was that before or after the move with your grandparents?”*	Supports approximate sequencing using familiar transitions rather than exact dates.	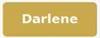
Cross-Domain Linking	*“What else was going on then — at school, at home, or with the court?”*	Encourages integration across education, family context, behavioral and psychiatric health, and juvenile justice involvement.	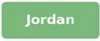
Substance Use Contextualization	*“How did alcohol or other substances fit into your life during that period?”*	Situates substance use within broader developmental and institutional contexts rather than as an isolated behavior.	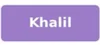
Clarifying Collapsed Memories	*“You mentioned a lot happening at once — can we slow that down and look at one piece at a time?”*	Helps differentiate overlapping or fused experiences without requiring chronological precision.	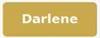
Participant Control Check	*“Would you like to stay here a bit longer, or move to another period?”*	Reinforces participant agency over pacing and depth of disclosure.	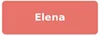
Distress Monitoring	*“We can pause or shift if this feels like too much.”*	Maintains trauma-informed boundaries and emotional safety throughout the interview.	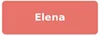
Closing / Integration	*“Looking back, how do you see these experiences fitting together now?”*	Supports meaning-making and reflective integration across domains and time.	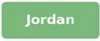

Note. Each prompt family is keyed to one or more vignettes in the Testimony in Practice section.

Prompts are illustrative; interviewer wording is adapted in real time to participant pacing, prior responses, and emotional state.

## STATEMENT OF PUBLIC HEALTH SIGNIFICANCE

The Anchored Testimony Calendar (ATC) is a Black feminist methodological tool for behavioral health research with structurally marginalized populations. ATC supports recall across nonlinear life courses, preserves participant narrative control, and surfaces the structural conditions that shape behavioral health—knowledge essential to designing equitable, responsive interventions.
