# Supplementary material for: The Anchored Testimony Calendar: A Black Feminist Method for Retrospective Behavioral Health Inquiry with Justice-Impacted Young Adults and Caregivers
Source: Behav Med. Author manuscript; Available in PMC 2026 Oct 23. (PMC13693432; doi:10.1080/08964289.2026.2683391)
Supplement: Supplementary file 1 [file NIHMS2197373-supplement-Supp_1.docx]

**Appendix 1. ATC Materials**

**A1.1 ATC Domains and Source Measures**

| **ATC Domain** | **Primary Constructs** | **Source Measures** |
| --- | --- | --- |
| **Behavioral & Psychiatric Health** | Psychiatric symptoms, substance use, healthcare access | DSM-5 Cross-Cutting Symptom Inventory; General Health Questionnaire; Adolescent Risk Behavior Assessment (ARBA); Behavior Assessment System for Children (BASC); Emotion Regulation Checklist |
| **Education** | School continuity, discipline, parent involvement | Demographics Questionnaire; Parent–Teacher Involvement Questionnaire (PTIQ) |
| **Family Context** | Communication, monitoring, conflict, caregiver stress | Family Assessment Device (FAD); Parent–Adolescent Communication (PAC); Parental Monitoring Questionnaire (PMQ); Stress Index for Parents of Adolescents (SIPA); Conflict Behavior Questionnaire (CBQ) |
| **Juvenile Justice Involvement** | Arrest, supervision, detention | Arrest & Treatment History Questionnaire; juvenile-justice items from the EPICC adolescent battery |

*Note. Cross-domain measures (Everyday Discrimination Scale; Neighborhood Environmental Scale; Cultural Stress Scale; YRBS items; peer-relationship and dating-violence items) populate cells across multiple ATC domains as contextually relevant. Full instrument citations available on request.*

**A1.2 Young Adult ATC Interview Guide (T8)**

*Each prompt covers the period from T1 (first EPICC participation) to the present. Prompts are interviewer-facing and follow participant pacing; the calendar is not shown to the young adult.*

1. **Eligibility anchor.** What event or circumstances brought you into EPICC at T1?
2. **Health.** What was going on in your life then — physical, mental, emotional? How has your health been since?
3. **Housing.** What was your living situation? What has housing looked like since?
4. **Relationships.** Were there relationships that mattered to you then? How would you characterize them — and how about since?
5. **Substance use.** Were you using any drugs (illicit or prescribed) then? How about since?
6. **Legal involvement.** Were you involved in any illegal activity then? How about since?
7. **Sexual activity.** Were you engaged in any sexual activity then — including anything you'd characterize as unsafe? How about since?
8. **Supports and resources.** What supports met your basic needs? What couldn't you access? How has that looked over time?

*In-vivo follow-up probes. The interviewer listens for participant talk about macro-level forces — racism, sexism, economic inequality — and names those forces only if the participant has named them first. Probes circle three lived sites where macro forces become legible:*

- **Identity —** "How were you seeing yourself at that time? Did how you saw yourself change after that?"
- **Treatment by others —** "How did people respond to you in that situation? What did you make of how they treated you?"
- **System navigation —** "What was it like dealing with school, the court, the clinic, or the agency then? What did you have to do to get through it?"

**A1.3 Caregiver ATC Interview Guide (T8)**

*Prompts invite reflection on both the caregiver's experience and the young adult's, from T1 (first EPICC participation) to the present.*

1. **Eligibility anchor.** What event or circumstances brought your young adult into EPICC at T1?
2. **Health.** What was going on in your lives then — yours and theirs? How have things been since?
3. **Living situation.** Were you living together? How has the household shifted since?
4. **Relationships.** What relationships mattered at the time, for you and for your young adult? How about since?
5. **Substance use.** Was either of you using drugs (illicit or prescribed) then? How about since?
6. **Legal involvement.** Was either of you involved in any illegal activity? How about since?
7. **Supports and resources.** What supports helped you and the young adult? What couldn't either of you access — religious community, social worker, health insurance, sobriety support, financial autonomy, sibling support?

*In-vivo follow-up probes. The interviewer listens for caregiver talk about macro-level forces — racism, sexism, economic inequality — and names those forces only if the caregiver has named them first. Probes circle three lived sites where macro forces become legible:*

- **Identity —** "How were you seeing yourself — and your young adult — at that time? Did either of those views shift after that?"
- **Treatment by others —** "How did people respond to you, or to your young adult, in that situation? What did you make of how they treated either of you?"
- **System navigation —** "What was it like dealing with school, the court, the clinic, or the agency for either of you then? What did you have to do to get through it?"
